# Renewable Carbonaceous Materials from Biomass in Catalytic Processes: A Review

**DOI:** 10.3390/ma17030565

**Published:** 2024-01-25

**Authors:** Juan J. Villora-Picó, Judith González-Arias, Francisco M. Baena-Moreno, Tomás R. Reina

**Affiliations:** 1 Inorganic Chemistry Department and Materials Sciences Institute, University of Seville-CSIC, 41092 Seville, Spain; jvillora@us.es (J.J.V.-P.); tramirez@us.es (T.R.R.); 2 Chemical and Environmental Engineering Department, Technical School of Engineering, University of Seville, C/Camino de los Descubrimientos s/n, 41092 Sevilla, Spain

**Keywords:** renewable carbonaceous materials, biomass, catalytic processes, green materials, green processes

## Abstract

This review paper delves into the diverse ways in which carbonaceous resources, sourced from renewable and sustainable origins, can be used in catalytic processes. Renewable carbonaceous materials that come from biomass-derived and waste feedstocks are key to developing more sustainable processes by replacing traditional carbon-based materials. By examining the potential of these renewable carbonaceous materials, this review aims to shed light on their significance in fostering environmentally conscious and sustainable practices within the realm of catalysis. The more important applications identified are biofuel production, tar removal, chemical production, photocatalytic systems, microbial fuel cell electrodes, and oxidation applications. Regarding biofuel production, biochar-supported catalysts have proved to be able to achieve biodiesel production with yields exceeding 70%. Furthermore, hydrochars and activated carbons derived from diverse biomass sources have demonstrated significant tar removal efficiency. For instance, rice husk char exhibited an increased BET surface area from 2.2 m^2^/g to 141 m^2^/g after pyrolysis at 600 °C, showcasing its effectiveness in adsorbing phenol and light aromatic hydrocarbons. Concerning chemical production and the oxidation of alcohols, the influence of biochar quantity and pre-calcination temperature on catalytic performance has been proven, achieving selectivity toward benzaldehyde exceeding 70%.

## 1. Introduction

During the last decades, the demand for carbon-based materials has intensified [1] due to the need for many economies to be developed. The highest share of carbon-based materials comes from non-renewable and traditional fossil carbon [2]. At this pace, this fact will cause not only a lack of carbonaceous materials but also serious environmental challenges. As the need for sustainable practices increases in society, the use of renewable carbonaceous materials is key. The inherent properties of renewable carbonaceous materials, such as low footprint and diverse chemical functionalities, make them uniquely suited for replacing traditional carbon-based materials [3]. Furthermore, the use of renewable carbonaceous materials aligns with the principles of green chemistry, emphasizing the design of processes that minimize environmental impact while maximizing efficiency.

Within the many potential applications for renewable carbonaceous materials, its integration into catalytic processes has emerged as a compelling solution with far-reaching implications [4,5]. Catalysis, as a pivotal technology in the chemical industry, offers a unique opportunity to address the pressing need for cleaner and more sustainable methodologies. The escalating depletion of finite fossil fuel resources and the associated environmental impacts of traditional catalytic processes underscore the urgency of transitioning toward renewable alternatives. By incorporating renewable carbonaceous materials derived from biomass, agricultural residues, and other sustainable sources into catalytic processes, our society can achieve a double aim. It can not only mitigate the environmental footprint associated with catalytic processes but also contribute to the development of a circular and sustainable economy.

There are some previous works dealing with the application of renewable carbon materials in catalytic processes. Some examples of these works are explained next. Biochar stands out as a highly promising catalyst and support for the process of biomass gasification. In the pursuit of efficient hydrogen production from biomass steam gasification, both biochar and Ni-based biochar were investigated. As a point of reference, commercial activated carbon was also included in this study for comparative analysis [6]. The investigation delved into the impact of various parameters such as gasification temperature, steam-to-biomass ratio, Ni loading, and biochar properties on the catalyst’s activity, specifically in terms of hydrogen production. Notably, the Ni/AC catalyst demonstrated superior performance at a gasification temperature of 800 °C, a steam-to-biomass ratio of 4, and a Ni loading of 15 wt.% [6]. Among the various tested catalysts, it was observed that Ni supported on cotton char exhibited the highest activity in hydrogen production, achieving a remarkable 64.02 vol.% and 92.08 mg/g biomass.

Another promising strategy for optimizing the utilization of oxygen within biomass resources is utilizing biomass catalytic pyrolysis products to derive valuable oxygen-containing chemicals [7]. The catalyst, playing a pivotal role in this process, needs to exhibit characteristics such as environmental friendliness, cost-effectiveness, high activity, and stability. In this line, a study conducted by Chen et al. introduced an innovative and environmentally sustainable method for producing phenols from bamboo waste via catalytic pyrolysis, employing N-doped biochar as the catalyst [7]. The investigation, conducted in a fixed bed reactor, aimed to unravel the catalytic pyrolysis mechanism facilitated by the N-doped biochar catalyst. The results underscored the significant enhancement of phenol generation, reaching an impressive 82% [7].

Biochar was also proposed as a key element to develop a catalyst for efficient production of biodiesel. Two solid acid catalysts based on carbon were synthesized by sulfonating pyrolysis char in two different ways, representing a total of four catalysts to be tested. The catalysts underwent comprehensive evaluation for their efficacy in catalyzing both the transesterification of vegetable oils and the esterification of free fatty acids. The comparison of the four resulting catalysts revealed that the catalyst with the highest surface area and acid density exhibited superior catalytic activity, particularly in the production of biodiesel from canola oil in the presence of methanol as the reagent. Additionally, the catalyst with a higher surface area demonstrated enhanced transesterification activity compared with catalysts with similar acid densities [8].

However, the works described are standalone studies dedicated to a reduced scope.

To close this information gap, this paper analyzes the main applications of carbonaceous materials in catalytic processes, as well as the innovative studies carried out so far by experts in this area. The main applications envisaged are biofuel production, tar removal, chemical production, photocatalytic systems, microbial fuel cell electrodes, and oxidation applications. This work is organized as follows. First, the production of the main renewable carbonaceous materials is explained, including the sources that they could come from and their properties. Later on, the different applications for catalytic processes identified are described in depth, including works carried out at different lab scales. We finalize the paper by concluding the main aspects of each application and the potential of renewable carbonaceous materials to transform catalytic processes into more sustainable activities.

## 2. Renewable Carbonaceous Materials from Biomass Production and Its Properties

Biomass, a sustainable and renewable material rich in carbon and primarily consisting of hemicellulose, cellulose, and lignin, has been commonly used as a raw material for crafting diverse high-value carbon-based products, including carbon materials, chemicals, and biofuels. Lignocellulosic biomass can typically be categorized into three primary components: cellulose, hemicellulose, and lignin [9]. Lignin-rich biomass is the most plentiful renewable carbon resource on Earth, second only to cellulose, boasting a global production of 40–50 million tons annually [10]. Given the high carbon content of lignin, lignocellulosic biomass emerges as a promising choice for use as a precursor in the production of valuable carbonaceous materials.

Currently, there is considerable focus on utilizing biomass as a cost-effective and environmentally friendly carbon source for generating valuable carbonaceous materials with different applications [11]. These high-value carbonaceous materials are primarily composed of pure carbon. In contrast, biomass contains not only carbon but also significant amounts of hydrogen, oxygen, nitrogen, and sulfur. Consequently, the essential steps for producing these materials from biomass involve breaking chemical bonds and eliminating hydrogen, oxygen, nitrogen, and sulfur, while preserving the carbon content.

Biomass conversion to valuable carbonaceous materials is mainly accomplished through various methods, including biological, chemical, and thermochemical processes. In general, thermochemical processes are favored for their shorter processing time, resulting in increased product yields [12]. Unlike biochemical processes that demand specific feedstock, thermochemical processes enable the utilization of the entire biomass to generate value-added materials [13]. Although thermochemical processing is the most used method to obtain carbonaceous materials from biomass waste since a carbonization step is required, it is worth noting that other alternatives are also used. Among them, chemical treatments such as the green activation method are also highlighted. In this case, the properties of a pre-carbonized product are improved using chemical activation [14]. While chemical activation is a widely used and effective technique for obtaining carbonaceous materials with desirable porous structures and high surface area, its broader application is hindered by drawbacks such as low yield and the need for substantial amounts of harmful activators and cleaning agents [14].

Among the different thermochemical processes for carbonaceous material production, pyrolysis, torrefaction, and hydrothermal carbonization (HTC) are highlighted [15]. During the thermochemical conversion, carbonization and graphitization of biomass take place regardless of the process. Below, a short overview of the most used processes for carbonaceous material production from biomass is shown.

### 2.1. Pyrolysis and Torrefaction

Pyrolysis and torrefaction of biomass involves breaking down polymer chains in biomass macromolecules through externally supplied heat under an inert atmosphere, resulting in the production of condensable volatiles (bio-oil), non-condensable gases, and a carbonaceous material commonly known as biochar. Torrefaction and pyrolysis are similar thermal processes employed in material conversion. Torrefaction operates within a lower temperature range (around 200–300 °C), while pyrolysis necessitates higher temperatures covering from 400 to 1000 °C. Notably, the differentiation between these processes primarily lies in the reaction temperature and the retention time of the material. Consequently, it is prevalent in the literature to encounter references to torrefaction as a form of slow- and mild-pyrolysis, emphasizing the shared characteristic of temperature-driven transformation while acknowledging the nuanced variations in their operational parameters [16,17]. The outcomes of these processes are influenced by the reaction conditions, with slow pyrolysis and torrefaction favoring low heating rates and long residence times for biochar production, while fast pyrolysis employs high heating rates and short residence times to predominantly generate bio-oil (up to 75%) [18]. The crude pyrolytic bio-oil can be further upgraded to advanced biofuels or bio-based chemicals. Numerous studies have explored biomass pyrolysis due to the potential of bio-oil and biochars [19,20,21]. Various pyrolysis techniques, such as co-pyrolysis, catalytic pyrolysis, microwave pyrolysis, and solar pyrolysis, have been developed to optimize and enhance pyrolysis processes for biomass conversion [22].

The composition of biomass plays a crucial role in shaping the outcomes of the pyrolysis/torrefaction process. As previously mentioned, biomass typically consists of cellulose, hemicellulose, and lignin, each contributing differently to the pyrolysis reactions [23]. Higher cellulose content in biomass has a notable effect on the pyrolysis process. It tends to accelerate the degradation of organic compounds during pyrolysis, leading to a faster release of volatile components. This acceleration results in an increased production of tar and gases. Therefore, biomass with a higher cellulose content tends to yield more tar and gases during pyrolysis. Conversely, lignin is a more intricate and resistant structure compared with cellulose, and its presence in biomass can slow down the pyrolysis process. The degradation of lignin requires more energy and time, contributing to a slower overall pyrolysis rate. Consequently, biomass with a high lignin content may yield lower amounts of tar and gases compared with biomass with higher cellulose content [24]. Furthermore, the type of biomass also has implications for the resulting char’s porosity. Different plant materials possess unique structural characteristics, and these variances influence the development of pores in the char produced during pyrolysis. For instance, research comparing char from various agriculture residues revealed that sugar cane bagasse and wood stem produced char with well-developed pores and high surface area, while others exhibited poor porosity development. This discrepancy in porosity has implications for the potential applications of the resulting char [25].

Additionally, the moisture content in biomass is a significant factor influencing the pyrolysis process. If the moisture content is high (above 30%), a considerable amount of the heat supplied during pyrolysis is utilized to remove moisture rather than facilitate the pyrolysis reactions. This situation slows down the heating rate, necessitating additional time to reach the target pyrolysis temperature. To optimize the pyrolysis process, especially for energy efficiency, it is advisable to use dry biomass or, if necessary, pre-dry the biomass to reduce moisture content before pyrolysis. This ensures that a larger portion of the heat is utilized for the intended thermochemical conversion, contributing to a more efficient and productive pyrolysis process [26].

Temperature, residence time, heating rate, and biomass particle size also play critical roles in shaping the formation of carbonaceous materials [27]. Higher temperatures during pyrolysis tend to reduce the yield of char but promote the devolatilization process, impacting the production of bio-oil and gases. Conversely, longer residence times and slower heating rates contribute to increased char yield, influencing the surface area and porosity of the resulting biochar [28,29].

Optimizing the pyrolysis process involves strategic considerations. Selecting dry biomass with a high lignin content is advisable, as it enhances energy efficiency during heating. Utilizing lower temperatures, slower heating rates, and longer residence times tends to maximize the overall yield of carbonaceous materials. However, it is noteworthy that higher temperatures and faster heating rates can enhance the porosity of carbonaceous materials, offering unique characteristics that may be desirable for certain applications [30].

### 2.2. Hydrothermal Carbonization

Unlike pyrolysis, HTC utilizes water as a solvent and reaction medium, allowing high-water-content biomass, eliminating the need for drying and reducing energy requirements [31]. However, hydrochars often exhibit low surface area and porosity, limiting their applications. The reaction mechanism involves hydrolysis, dehydration, decarboxylation, aromatization, and recondensation, with temperature influencing pathways. This process is primarily governed by hydrolysis [32]. In this step, hemicellulose, cellulose, and lignin undergo decomposition into smaller fragments, facilitating subsequent reactions like dehydration and decarboxylation. These reactions are crucial for reducing the H/C and O/C ratios, ultimately leading to the formation of a solid carbonaceous product. Further reactions, like decarboxylation, contribute to the degradation of carboxyl–carbonyl groups and the release of the main components of the final flue gas, primarily composed of CO_2_ and CO [32].

The characteristics of hydrochar produced through HTC are intricately influenced by the composition of the biomass, the specific type of biomass utilized, and the operating conditions employed during the HTC process [33]. The complex nature of lignin makes it less degradable, contributing to the overall mass of hydrochar produced during the HTC process [34].

The morphological changes in biomass during HTC are notably influenced by the temperature at which the process is conducted. Lower temperatures within the range of 150–200 °C are conducive to maximizing the production of solid hydrochar [33]. In contrast, higher temperatures lead to extensive dehydration, resulting in a reduction in hydrochar yield [35]. This temperature-dependent morphological alteration is crucial in determining the structure and properties of the resulting hydrochar. Furthermore, temperature not only affects yield but also influences the surface area of the hydrochar [36]. Higher temperatures during HTC tend to yield hydrochar with larger surface areas. This is a critical aspect as the surface area impacts the reactivity and applicability of hydrochar in various applications, such as soil amendments or electrochemical devices.

The residence time also plays a pivotal role. Longer residence times, especially at lower temperatures, enhance hydrochar yield. This prolonged exposure allows for more extensive carbonization and recondensation reactions, contributing to the overall yield and characteristics of the hydrochar [37].

It is worth noting that the HTC process itself is relatively slow, ranging from hours to days [38]. The slow kinetics of the process allow for careful control and manipulation of the reactions, contributing to the versatility of HTC as an approach for biomass conversion. The ability to tailor hydrochar characteristics by adjusting parameters such as temperature, residence time, and biomass composition makes HTC a versatile and adaptable method for transforming biomass into functionalized carbonaceous materials for different applications.

In contrast to carbonaceous materials produced via thermal pyrolysis, HTC materials feature furanic and aromatic units containing oxygen-substituted arene-type moieties. This characteristic opens up the potential for incorporating supplementary substances during the hydrothermal reaction or through post-functionalization, thereby allowing for the further adjustment of the physical and chemical attributes of HTC materials [39].

Until now, we have focused on the most used thermochemical processes to produce renewable carbonaceous materials. This is a key point to understand their potential for catalytic processes. Nonetheless, the readers could benefit from understanding where carbonaceous materials are used nowadays. Therefore, the next section presents a brief overview of their present applications before diving into the catalytic applications of renewable carbonaceous materials.

### 2.3. Overview of Applications for Carbonaceous Materials

After explaining the different thermochemical processes used in the production of renewable carbonaceous materials along with their respective properties, it is worth highlighting the present utilization of carbonaceous materials before moving to their potential catalytic applications. Indeed, carbonaceous materials are not new to our society. These materials have a long history in synthesis and applications and are integral to daily life. Examples include activated carbon for deodorization and water purification, graphite in pencils, carbon fibers for sports equipment, and carbon black in inks and pigments. The accidental discovery of synthetic graphite in the 19th century led to the development of various carbon materials like glass-like carbon and carbon fibers, widely applied in industry [40].

As shown above, biochar, the most well-established carbonaceous material derived from biomass, is produced via the thermochemical transformation of organic matter. Moreover, activated carbon and carbon fibers, both originating from biomass, have been formulated for diverse applications [41,42,43]. The exploration of innovative carbon-based nanomaterials, such as fullerene, carbon nanotubes, graphene, and graphene quantum dots, has significantly attracted interest in the creation of carbon nanostructures from carbonaceous materials derived from biomass [44]. In Table 1, we group a list of works dealing with different carbonaceous materials obtained from various biomass waste. This serves as a summary to show the wide variety of applications of these materials. Table 1 not only provides a comprehensive overview of different carbonaceous materials but also outlines the various applications they serve. The thermochemical processes involved in obtaining these materials are also presented in Table 1.

## 3. Applications of Carbonaceous Materials in Catalytic Processes

### 3.1. Biofuel Production

**Figure 1:**
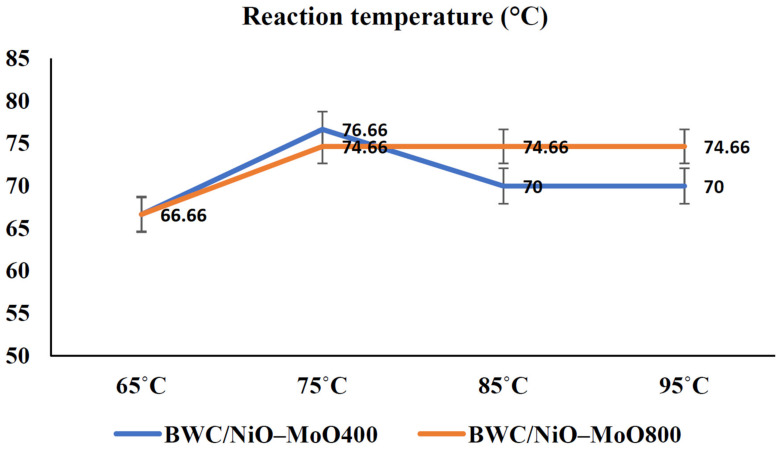
Biodiesel yield (%) obtained at various reaction temperatures (°C) of biochar woodchip. Reproduced from [71].

The escalating demand for energy resources, propelled by population expansion and economic advancement, necessitates the exploration of alternatives to existing fuels characterized by finite resources and environmental ramifications such as the greenhouse effect and climate change [66]. In light of these considerations, biofuels emerge as a viable alternative, with biodiesel standing out as the most widely consumed biofuel globally [67]. A predominant methodology for biodiesel production involves the transesterification of used cooking oil, wherein the oil undergoes a chemical reaction with methanol [68]. While homogeneous alkaline catalysts like KOH or carbonates are frequently employed, their limited recoverability prompts a quest for heterogeneous catalysts [69]. Catalysts encompass alkali metal hydroxides, metal oxides, or zeolites [70], yet an imperative focus remains on identifying economically favorable catalysts. Utilizing carbonaceous materials as supports, particularly biochar, is a judicious choice owing to their economic viability and apt chemical and textural characteristics. In this context, Azman et al. systematically synthesized nickel oxide (NiO) and molybdenum oxide (MoO) catalysts, employing a biochar derived from wood chips as a robust support matrix. The catalytic performance was assessed, revealing yields exceeding 70% at an operational temperature of 75 °C for the catalyst subjected to calcination at 400 °C (Figure 1). This experimental outcome underscores the catalytic efficacy of the prepared NiO and MoO catalysts, as supported on the designated biochar substrate, within the specified reaction conditions [71]. CaO commands significant attention due to its commendable catalytic activity, cost-effectiveness, and robust basicity. Di Bitonto et al. demonstrated the preparation of CaO catalysts supported on biochar derived from avocado seeds, yielding remarkable conversions of 91% [72]. The potential concern of CaO leaching into the reaction medium underscores the necessity for stability promoters, often rooted in mixed oxides such as SiO_2_ [73]. Nevertheless, certain biochars exhibit inherent catalytic activity, obviating the need for additional support materials. Daimary et al. conducted a study wherein biochars with elevated concentrations of alkali and alkaline earth metals, primarily potassium, were derived from potato skins. The catalytic outcomes indicated exceptional activity, with conversion values surpassing 97% [74]. Alkaline catalysts, however, are susceptible to saponification, complicating the separation of reaction products. An intriguing avenue involves the incorporation of –SO_3_H groups onto biochar surfaces, as these groups have demonstrated catalytic prowess in transesterification processes. Yadav et al. achieved yields exceeding 99% with excellent recyclability by subjecting biochars from bamboo and coconut husk to post-treatment with H_2_SO_4_ [75]. Analogous outcomes are observed with biochars sourced from olive pits, nut shells, microalgae, or discarded cork [67,76].

The Fischer–Tropsch synthesis (FTS) process constitutes a prominent method for the catalytic conversion of synthesis gas into hydrocarbons and oxygenated compounds, with a primary focus on optimizing yield for the production of light olefins suitable for use as fuels [77]. The most commonly used catalyst is Co and other metals used as promoters (Ru, Ni, and Fe) supported on metal oxides (e.g., Al_2_O_3_, TiO_2_, or SiO_2_) [78]. However, challenges arise with certain metals, notably Co, which can form irreducible compounds like CoAl_2_O_4_, leading to rapid catalyst deactivation. Biochars present an intriguing alternative owing to their favorable porous properties and elevated thermal stability. Yousefian et al. demonstrated the preparation of biochars derived from rice husk, coconut husk, and algae as supports for cobalt catalysts, showcasing the capacity of carbonaceous materials to enhance Co reducibility. The catalytic outcomes proved highly satisfactory, achieving a CO conversion rate of 67% [79]. Surface chemistry modifications on biochar significantly influence catalytic activity, as exemplified by Bai et al. who introduced nitrogen through urea doping. This alteration modified the electronic structure of the material, enhancing metal dispersion and metal–support interaction, resulting in conversion rates exceeding 90%, with selectivity toward heavier hydrocarbons reaching approximately 50% [80]. Exploring Fe as a catalyst is noteworthy due to its heightened selectivity toward olefins and its inherent water–gas shift activity, thereby augmenting hydrogen content in the feed. Teimouri et al. undertook the preparation of Fe catalysts on biochar derived from canola hulls, achieving a selectivity exceeding 72% toward C_5_+ products [81]. This diversification in catalyst materials and surface modifications on biochar underscores the dynamic nature of catalyst development within the FTS process, with ongoing efforts aimed at enhancing efficiency and selectivity.

Hydrogen, characterized by its high energy content and environmental friendliness, is regarded as a pivotal fuel in the paradigm of future energy sources [82,83]. Various processes can yield hydrogen, with dry reforming of methane emerging as a prominent industrial method. This reaction facilitates the conversion of two major greenhouse gases, CO_2_ and CH_4_, into H_2_. Carbon-based catalysts have garnered substantial attention due to their inherent stability and resistance to deactivation by sulfur compounds. In a study conducted by Zhao et al., catalysts derived from biochar sourced from hawthorn seeds exhibited exceptional performance following torrefaction treatment, achieving conversions surpassing 70% [84]. Despite the efficacy of existing methods, advancements have introduced novel approaches, such as the catalytic decomposition of methane. In this context, surface carboxylic groups on biochar act as catalysts for methane decomposition. However, the formation of carbonaceous deposits results in the rapid deactivation of the catalyst, necessitating comprehensive strategies to address this challenge [85]. This predicament underscores the ongoing efforts in the scientific community to innovate and refine processes, ensuring the sustained viability and efficiency of biochar-based catalysts in the production of hydrogen, a critical component of the envisioned clean energy landscape.

In Table 2, we summarized the various biomass-derived catalysts for biofuel synthesis.

In conclusion, the global demand for sustainable energy alternatives has fueled extensive research in biofuels, particularly biodiesel production. The use of carbonaceous materials, especially biochar, as catalyst supports has shown economic viability and technological promise. Future research should focus on optimizing biochar-supported catalyst synthesis processes and addressing stability concerns. In the FTS, biochar-supported catalysts offer improved reducibility and enhanced selectivity, showcasing dynamic developments in catalyst materials. Looking forward, hydrogen production through processes like dry reforming of methane using biochar-based catalysts is a key focus. Despite promising results, ongoing research is needed to overcome challenges such as catalyst deactivation and ensure sustained efficiency in the evolving landscape of clean energy production. As the scientific community continues to innovate, these findings contribute to the sustainable development of biochar-based catalysts, paving the way for a cleaner and more efficient energy future.

### 3.2. Tar Removal

Due to the growing interest in alternative energy sources caused by increased CO_2_ emissions and global climate concerns, biomass is seen as a renewable energy source, with gasification being a promising technology for biomass-to-energy conversion. Gasification, while effective, faces challenges, especially in tar removal. Two main groups of methods (i.e., in situ and downstream) aim to address tar issues [86,87]. The use of carbonaceous materials is proposed as a potential solution for downstream tar removal [88,89,90].

The adsorption capabilities of carbonaceous materials for contaminants are attributed to their distribution of porous structures, high specific surface areas, and enhanced surface chemistry properties. The effectiveness of adsorption also depends on factors like hydrophobicity, alkalinity, ion exchange capacity, and elemental composition. As an example, hydrochars prepared at low temperatures (e.g., 180 °C) without additional heat treatment and/or surface activation initially possess a limited pore structure. However, oxygen groups on the surface promote adsorption and enable further surface modification [91].

As already mentioned, the most important structural characteristic of the carbonaceous materials to be used as tar adsorbents is having a high surface area measured by the distribution of micropores, mesopores, and macropores. The chemical characteristics are determined by the presence of inorganics scattered on the surface and oxygen-containing functional groups at the borders of the graphite sheets. These oxygen sites enhance the adsorption of polar molecules, influencing the adsorption capacity and catalytic activity of the carbonaceous material [92,93].

**Figure 2:**
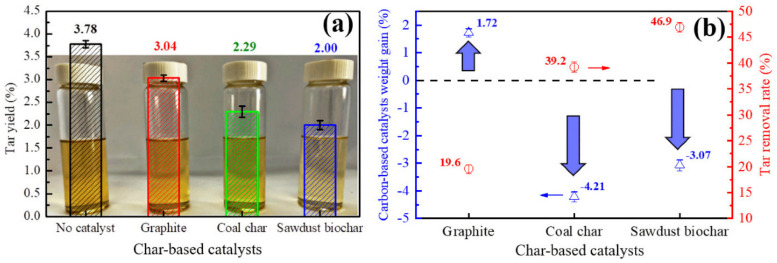
The results of tar reforming over the three selected carbon materials. (**a**) Tar yields measured; (**b**) weight gain and the rate of tar reforming. Reproduced from [94].

Numerous studies have highlighted the effectiveness of carbonaceous materials obtained through thermal treatments in the removal of tars in industrial applications. For instance, research has demonstrated the enhanced adsorption capacity of activated carbons derived from wood waste in gasification systems. In pyrolysis processes, a significant reduction in tars has been observed using porous carbons derived from agricultural residues. Zhang et al. demonstrated the effectiveness of different carbonaceous-based materials for in situ tar reforming in their work. They observed a significant reduction in the tar yield when using the different materials, resulting in great tar removal rates ranging from ca. 20% to ca. 47% [94]. The results that they obtained can be graphically seen in Figure 2.

Similarly, activated carbons find extensive application in adsorption processes, aiding in the capture of organic volatile compounds and adsorbing hydrocarbons and PAHs [95,96,97]. Additionally, activated carbons are frequently employed in gas cleaning within combustion plants, effectively eliminating metals and dioxins at temperatures ranging from 150 to 200 °C [98].

Examples of different carbonaceous materials used as tar adsorbents can be found in the literature as shown below. Liu et al. assessed rice husk and corncob biochar adsorption of phenol. The results show a significant increase in adsorption capacities at phenol concentrations from 5000 to 8000 mg/L, with stability at concentrations exceeding 8000 mg/L. Biochar adsorption is influenced by both surface areas and total functional group contents, which is impacted by retention time during the two stages of pyrolysis and carbonization [99].

Phuphuakrat et al. introduced a two-step method for tar removal using decomposition and adsorption. Activated carbon demonstrated superior adsorption of light aromatic hydrocarbon tars and light PAH tars compared with wood chips and synthetic porous cordierite. Wood chips, however, proved practical by effectively minimizing condensable tar without compromising system efficiency, making it a noteworthy tar adsorbent [100].

**Figure 3:**
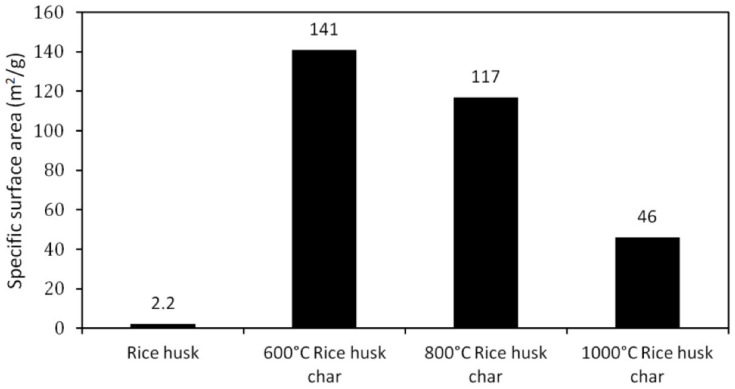
Specific surface area values for the BET analysis of rice husk and rice husk chars. Reproduced from [101].

Similarly, Paethanom et al. investigated the adsorption capabilities of rice husk char as a tar adsorbent, which exhibited a positive adsorption performance. Following thermal decomposition at elevated temperatures, the pores of the rice husk char were well developed, leading to an increased surface area. For example, raw rice husk had a low BET surface area of 2.2 m^2^/g, while the pyrolyzed char at 600 °C exhibited a higher BET surface area of 141 m^2^/g. Nonetheless, increasing the temperature led to a reduction in the BET surface area [101]. This can be observed in Figure 3, showing the great importance of pyrolysis temperature on the final adsorption capabilities of the carbonaceous materials.

Heavy tar components could condense at the bottom of the char bed, suggesting that tar removal using rice husk char involved both tar molecule adsorption in the char material’s pores and condensation of heavy tar molecules in the hot gas as it passed through the adsorption bed at ambient temperature. Although some char was soaked by the condensed heavy tar, the majority of the rice husk char remained dry after tar adsorption. Consequently, the char adsorbent is suitable for dry gas light tar adsorption, extending its service life. Effective adsorption of light aromatic hydrocarbon tars like xylene and styrene was also observed. However, subsequent poor adsorption performance and increased tar amounts were noted due to de-adsorbed tars surging out with the exit gas. Phenol removal was also notably successful in this work [101,102]. For large-scale applications, it is advised to assess the saturation point of biochar adsorbent under specific operating conditions before gas cleaning system design. Various methods, including thermal, extractive, and chemical regeneration processes, can be employed to treat or regenerate waste adsorbents in general [103,104].

In terms of tar conversion, carbonaceous materials are also a good option to consider. For example, El-Rub et al. showed a comparison of biomass char to various catalysts like dolomite and nickel during biomass gasification at a temperature range of 700–900 °C in their work. They used phenol and naphthalene as model tar compounds. Dolomite and nickel showed the highest phenol conversion at 90% and 91%, respectively. Their reforming capabilities were evident, producing H_2_ and CO. Biomass-derived char exhibited moderate phenol conversion (i.e., 82%) due to thermal cracking at high gasification temperatures. Despite a limited lifetime, continuous activation through gasification reactions enhances char stability. Nickel catalyst excelled in naphthalene removal, while the lower conversion of dolomite was linked to its low iron content. Biomass chars, excluding nickel, displayed high activity, offering stability and effectiveness compared with the sensitive nickel catalyst [105].

Additionally, Fuentes-Cano et al. explored the catalytic decomposition of two model tars (toluene and naphthalene) using three distinct carbonaceous materials (coconut char, coal char, and dried sewage sludge char). The primary mechanisms in tar conversion over carbonaceous material involve deposition, dehydrogenation (resulting in soot formation on the char surface), and soot gasification, mirroring the processes observed in tar conversion over porous particles [106].

The catalytic performance of char is influenced by various factors, including biomass resources, gasification/pyrolysis conditions, catalytic conditions, gasifier types, and tar composition. Klinghoffer et al. extensively discussed the use of residual char as a catalyst for tar conversion, emphasizing its dependence on gasification temperature and time. Char samples from gasification at 550 °C showed inadequate BET surface area due to high residual organics. However, increasing reaction time or temperature led to higher surface area chars, ranging from 429 to 687 m^2^/g [107].

In conclusion, the utilization of carbonaceous materials for tar removal and conversion presents a promising avenue for advancing sustainable energy production. The adsorption capabilities of these materials, attributed to their porous structures and surface chemistry properties, have been demonstrated in various studies to effectively address the challenges associated with tar removal. Future research endeavors could focus on optimizing the synthesis methods to enhance the structural characteristics, such as surface area and pore distribution, thereby improving adsorption capacities. Additionally, exploring innovative approaches for in situ tar reforming and investigating the long-term stability of carbonaceous materials in large-scale applications would contribute valuable insights. Furthermore, the catalytic potential of biomass-derived chars warrants further investigation, emphasizing the influence of various factors on their performance in tar conversion processes. By addressing these aspects, future research can contribute significantly to the development of efficient and sustainable biomass-to-energy conversion technologies.

### 3.3. Chemical Production

**Figure 4:**
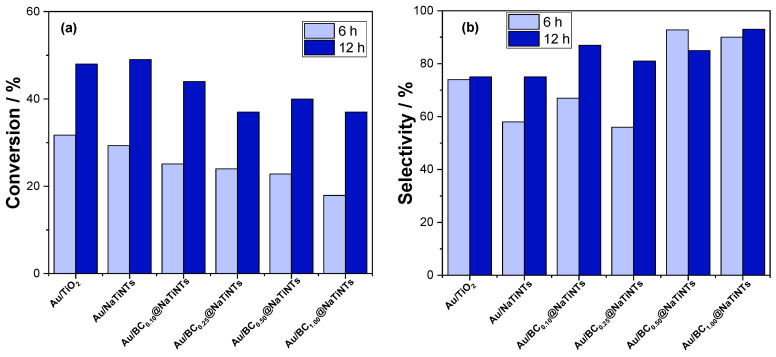
The conversion (**a**) and selectivity (**b**) of 6 h and 12 h oxidation reactions of air-calcined catalysts at 400 °C. Reproduced from [108].

Biochar finds significant application in the synthesis of high-value compounds, serving both as a catalyst support and as a metal-free catalyst. One notable application is in the oxidation of alcohols, a crucial process in industries such as pharmaceuticals, petrochemicals, and plastics. Chen et al. undertook the preparation of carbon materials derived from dried grain residues obtained from distilleries, aiming to modify titanate nanofibers with supported gold nanoparticles. The study meticulously investigated the influence of biochar quantity and the temperature of pre-calcination treatments on the catalytic performance of the material. Furthermore, the incorporation of biochar demonstrated enhanced control over selectivity, particularly directing the reaction toward benzaldehyde, a challenging aspect in alcohol oxidation reactions (Figure 4) [108]. Catalytic oxidation of carbonyl compounds is equally pivotal in chemical and drug synthesis. Abedian-Dehaghani et al. employed biochars co-doped with selenium and nitrogen for aldehyde oxidation, achieving yields exceeding 90% [109]. Biochars also play a crucial role in diverse processes, including the oxidative dehydrogenation of N-heterocycles using air as an oxidant. Pang et al. successfully prepared biochars from wheat husk, attaining yields surpassing 90%. The high hydrophobicity of the biochar proved advantageous in facilitating the removal of produced water from active sites [110].

Hydrogenation reactions represent crucial processes in industrial applications, and biochars emerge as viable catalysts in such reactions. Longo et al. employed Pd catalysts supported by biochars for the hydrogenation of benzaldehyde to yield toluene. Utilizing diverse biochars sourced from both animal and plant biomass, the catalytic results demonstrated high efficacy, achieving total conversion within 2 h of reaction time and displaying selectivities exceeding 95%, accompanied by commendable recyclability [111]. The reduction of nitroarenes is another industrially significant reaction, serving as a primary route to produce anilines, crucial precursors for drugs or dyes [112]. In this regard, Kumar et al. developed a RuO_2_ catalyst supported by biochar derived from pine needles. The catalysts exhibited elevated conversion values attributed to the presence of nitrogen within the carbonaceous network, facilitating improved distribution and stability of Ru nanoparticles [113]. Sadjadi et al. synthesized Pt catalysts supported on biochar derived from a specific plant, achieving high conversion values exceeding 97%. However, due to complications in recovery, they incorporated Fe_3_O_4_ to confer magnetic properties, thereby enhancing recyclability [114]. Additionally, they prepared a biochar–halosite nanocomposite, serving as a support for Pd catalysts, yielding satisfactory conversion results [115]. The surface chemistry of biochars plays a pivotal role in the catalytic activity of the resultant materials, as surface groups have been identified to contribute to the stabilization of metal nanoparticles [116]. Ren et al. prepared biochars with a high nitrogen content from sewage sludge, demonstrating significant catalytic activity in the reduction of nitrophenols with a remarkable 90% conversion within a 4 min reaction time, coupled with robust recyclability. In this context, carbon atoms adjacent to nitrogen sites serve as crucial reaction sites [117]. Moreover, the presence of oxygen or nitrogen groups influences the hydrophilicity of the carbonaceous material, thereby enhancing chemical activity. This was explored by Wang et al., who investigated the impact of oxygenated groups in the hydrogenation of organic compounds (eugenol, vanillin, and nitrobenzene). The results revealed that nitrobenzene, lacking strong hydrogen bonds, was less affected by changes in polarity, while vanillin and eugenol demonstrated facilitated activation of the (C=O) and (C=C) groups, respectively [118].

**Figure 5:**
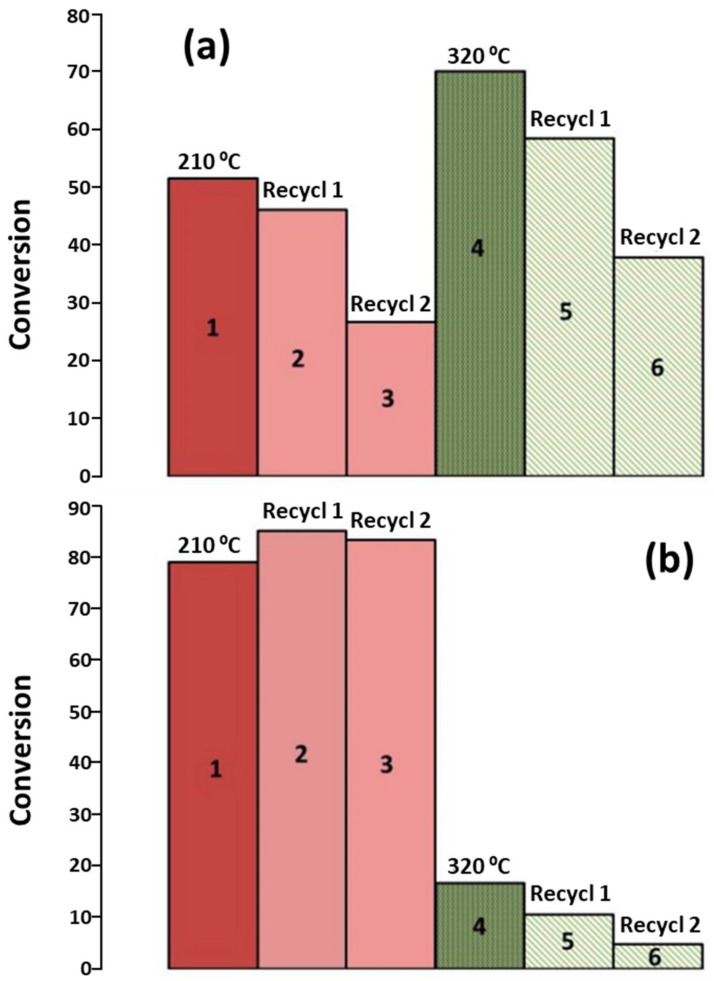
(**a**) Conversion and (**b**) selectivity to FA at 210 °C (columns 1–3) and at 320 °C (columns 4–6). Reproduced from [122].

The valorization of biomass for the production of high-value-added chemicals stands as a significant application of biochars. Wang et al. engineered a Ni-Mo_2_C catalyst supported on a graphitized biochar, derived from sawdust, for the hydrogenation of lignin. The incorporation of the metal catalyst into the biochar elevated the yield of liquid products to over 60%, demonstrating remarkable recyclability. This catalytic efficacy is attributed to the synergistic interaction between biochar and metal nanoparticles, facilitating efficient electron transfer [119]. In a parallel investigation, Zhang et al. focused on the microwave-assisted oxidation of biomass-derived glucose to produce gluconic acid (GUA) and glucuronic acid (GOA). Employing Cu catalysts supported by biochars, they achieved yields of 39.0% and 30.7% for GUA and GOA, respectively. These favorable catalytic outcomes are ascribed to the high percentage of Cu and Cu_2_O species, along with a substantial presence of oxygenated functionalities within the biochar matrix [120]. Catalytic hydrogenation of aldehydes holds significance as a stabilizing step in the conversion of bio-oils. Bardestani et al. explored the hydrogenation of furfural to obtain furfuryl alcohol (FA), a precursor for levulinic acid. Their study revealed conversions exceeding 50% and selectivities surpassing 90% by utilizing Ru supported on a somewhat oxidized biochar, which serves to stabilize the Ru nanoparticles [121]. Fuente-Hernandez et al. synthesized Pt catalysts supported on biochar derived from maple biomass. The results exhibited conversions up to 50% at 210 °C, with a selectivity of over 80% toward the desired product (Figure 5) [122].

In conclusion, biochar serves as a versatile catalyst and support, with notable applications in alcohol oxidation, aldehyde oxidation, and hydrogenation reactions. The incorporation of biochar in these processes enhances selectivity and catalytic efficiency. Future research could focus on optimizing biochar synthesis for specific surface functionalities and exploring sustainable recovery methods for catalysts. The valorization of biomass using biochar-supported catalysts for high-value chemicals, such as lignin hydrogenation and glucose oxidation, demonstrates the potential of biochar in sustainable chemical production. Further exploration in stabilizing steps, like the hydrogenation of furfural, presents opportunities for advancement. As the field progresses, a deeper understanding of biochar–catalyst interactions will contribute to the sustainable development of biochar-based processes for high-value chemical synthesis.

### 3.4. Photocatalytic Systems

The discipline of photocatalysis is centered on the advancement of chemical reactions facilitated by the presence of light and a photocatalyst, which, in turn, augments the reaction rate under light conditions [123]. A prominent application within the realm of photocatalysis is the remediation of pollutants from wastewater, encompassing both organic [124] and inorganic pollutants [125]. Titanium dioxide (TiO_2_) stands out as the catalyst of choice due to its widespread usage, attributed to its cost-effectiveness, facile synthesis, and robust stability. However, a prominent challenge in its application lies in the substantial bandgap (3.2 eV), restricting its responsiveness solely to ultraviolet light [126]. Additionally, TiO_2_ presents an additional impediment concerning the swift recombination of charge carriers, thereby impeding radical formation [127]. Nevertheless, research has demonstrated that the incorporation of support alongside TiO_2_ can yield advantageous outcomes. Recent investigations have particularly highlighted the intriguing potential of utilizing biochars as a supporting material, considering economic, environmental, and photocatalytic perspectives. Notably, the deployment of TiO_2_–biochar composites has been observed in multiple studies to impede electron–hole recombination, thereby substantially enhancing the catalytic activity of the composite material [128].

**Figure 6:**
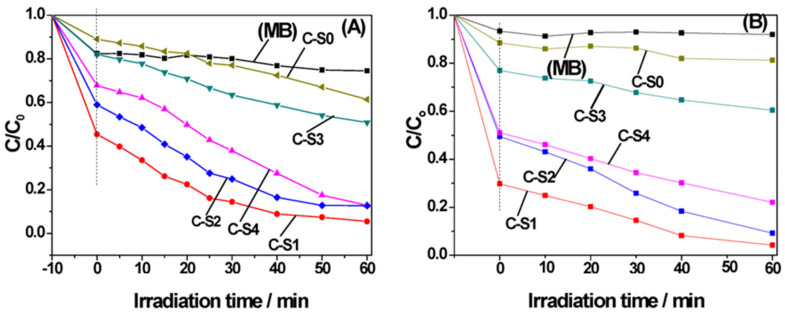
Photocatalytic degradation of MB using the samples (**A**) under UV irradiation and (**B**) under visible irradiation. Reproduced from [131].

Moreover, the utilization of this material category facilitates a reduction in the bandgap of the photocatalyst, enabling enhanced utilization of visible radiation, as substantiated by the findings of Wang et al. in their investigation of ammonium removal from aqueous solutions. Their study demonstrated satisfactory outcomes achieved under simulated sunlight conditions [129]. The choice of biochar is a critical determinant in the photocatalytic performance of the materials, encompassing both physical attributes such as BET surface area, porosity, and conductivity, as well as chemical characteristics. Hou et al. undertook the preparation of TiO_2_–biochar catalysts employing two distinct biochar types: one characterized by a significant number of surface groups and another with a more limited abundance. Their assessment of catalytic efficacy in the removal of Cr(VI) from wastewater revealed that the biochar with an increased number of surface groups exhibited a proclivity for Cr(VI) adsorption and engendered a higher concentration of radicals under light exposure, thereby affecting a substantial enhancement in catalytic activity. Importantly, this study demonstrated the capability of the biochar sample with heightened surface groups to sequester electrons, thereby sustaining catalytic activity even subsequent to the cessation of the light source [130]. Similarly, Wang et al. prepare biochar/TiO_2_ composites using bamboo powder as a carbon source. The synergistic effects of carbon doping with TiO_2_ are investigated under visible and UV light (Figure 6). A decreasing effect of surface area is observed as the particle size increases. The variation in these parameters significantly influences the catalytic activity [131].

**Figure 7:**
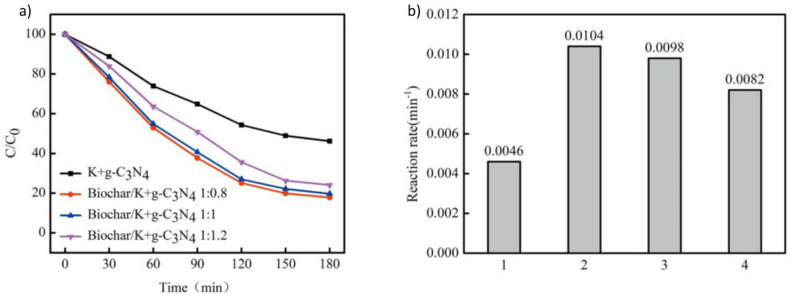
(**a**) Photocatalytic degradation curve of naphthalene and (**b**) the corresponding apparent rate constants (1—K + g-C_3_N_4_; 2—biochar/K + g-C_3_N_4_ 1:0.8; 3—biochar/K + g-C_3_N_4_ 1:1; and 4—biochar/K + g-C_3_N_4_ 1:1.2). Reproduced from [135].

Nonetheless, analogous outcomes were replicated with alternative catalysts such as Cu_2_O or Bi_2_WO_6_ [132]. The adoption of single-phase photocatalysts is frequently constrained by their suboptimal responsiveness to visible light and a rapid recombination rate of electron–hole pairs, culminating in diminished catalytic activity [133]. The collaborative interplay between biochar and such photocatalysts, akin to TiO_2_, culminates in the fine-tuning of the bandgap, thereby enhancing their sensitivity to visible light and optimizing the utilization of photoinduced carriers [132]. An unconventional alternative to conventional semiconductors is graphitic carbon nitride (g-C_3_N_4_), prized for its cost-effectiveness. However, the expeditious recombination of electron–hole pairs and feeble responsiveness to visible light curtail its practical application. Nevertheless, the incorporation of g-C_3_N_4_ onto a biochar substrate affords a π-conjugated structure, thereby modulating charge separation efficiency, refining the optical properties of g-C_3_N_4_, and augmenting the specific surface area. This augmentation is exemplified by Meng et al.’s work, wherein the combination demonstrated notable efficacy in the degradation of rhodamine and methyl orange from water [134]. Indeed, the incorporation of K into g-C_3_N_4_ manifests an enhancement in its photocatalytic efficacy under visible light irradiation. This improvement is attributed not only to the alteration in its electronic structure but also to the heightened adsorption capacity resulting from the dopant introduction. Empirical validation of this phenomenon has been substantiated by Li et al. in the context of naphthalene removal (Figure 7) [135]. Furthermore, certain biochars manifest inherent photocatalytic activity in the absence of supplementary materials. This is particularly pertinent for biochars endowed with a profusion of oxygenated groups, behaving akin to semiconductors wherein electrons within defect zones (valence band) are stimulated by visible light, traversing toward oxygenated groups (conduction band). This transfer of electrons to dissolved oxygen induces the generation of -OH radicals, as elucidated in the research by Xiao et al. [136].

Furthermore, photocatalysis finds diverse applications in processes related to the conversion of carbon dioxide (CO_2_) into fuels or higher-value compounds [137,138], as well as in the environmentally clean generation of hydrogen through water splitting [139]. Similar to other applications, the utilization of single-phase photocatalysts often yields suboptimal catalytic activity, prompting interest in the modification of these materials through the incorporation of carbonaceous supports. In this context, biochars emerge as an economically advantageous alternative when compared with other carbonaceous materials such as graphene or carbon nanotubes. Notably, catalysts relying on titanium dioxide (TiO_2_), zinc germanate (ZnGe_2_O_4_), or carbon nitride, among others, have been explored, yet their efficiency remains distant from practical applications [140]. In a notable study, Yang et al. engineered Co-Al catalysts supported on biochar derived from cherry blossoms, yielding highly satisfactory results. This outcome suggests that the biochar can adeptly accommodate photoexcited electrons, thereby fostering the catalytic reduction of CO_2_ to CO [141]. Additionally, the hydrophilic or hydrophobic nature of the photocatalyst significantly influences the conversion outcomes, given that CO_2_ exhibits enhanced adsorption on hydrophobic materials. This challenge compounds the limitations of conventional metal oxide-based photocatalysts, primarily characterized by hydrophilicity. Consequently, the utilization of a hydrophobic material such as biochar confers an amphiphilic character to the catalyst, furnishing a more favorable surface for CO_2_ adsorption along with a hydrophilic component that enhances charge transfer [140]. Concerning water splitting, there exists noteworthy research wherein photocatalysts are developed through a synergistic combination of a semiconductor and biochar. For instance, Bhavani et al. conducted a study in which they prepared ZnIn_2_S_4_ catalysts supported on biochar sourced from rice husk, achieving highly intriguing catalytic results [142].

In summary, photocatalysis, a crucial field for chemical reactions under light, finds notable application in wastewater remediation and carbon dioxide conversion. Biochar, when used as a supporting material, addresses challenges associated with traditional photocatalysts. It enhances electron–hole separation, reduces the bandgap, and allows better utilization of visible light. Biochar-type choice and surface groups play crucial roles in its performance. Synergistic effects with other catalysts improve responsiveness to visible light. Certain biochars exhibit inherent photocatalytic activity, especially those with oxygenated groups. Biochar-supported catalysts show promise in converting carbon dioxide and environmentally clean hydrogen generation. Future research should optimize biochar properties, explore catalyst combinations, and understand the mechanisms for enhanced photocatalytic performance. The use of biochar in photocatalysis offers an economically advantageous and environmentally friendly alternative for sustainable applications.

### 3.5. Electrocatalysis

Fuel cells are devices aimed at converting chemical energy into electrical energy without combustion. In this process, fuels such as hydrogen undergo oxidation in the anode, producing electrons and either H^+^ or H_2_O in acidic and alkaline electrolytes, respectively. Subsequently, the electrons flow to the cathode, where they participate in the reduction of O_2_ to either H_2_O or OH^−^. This unique electrochemical process contributes to the higher energy efficiency and reduced environmental impact of fuel cells compared with traditional diesel or gas engines [143]. The efficiency and environmental benefits of fuel cells are inherently tied to their electrochemical processes, wherein hydrogen oxidation reaction (HER) at the anode and oxygen reduction reaction (ORR) at the cathode play crucial roles in converting chemical energy into electricity.

Cutting-edge electrocatalysts employed in ORR, oxygen evolution reaction (OER), and HER typically rely on precious metal-based materials such as platinum or its alloys for ORR and HER, and RuO_2_ or IrO_2_ for OER [144]. Despite their exceptional catalytic performance, these precious metal catalysts face significant drawbacks, including high cost and scarcity, making large-scale applications economically challenging. Moreover, these materials encounter stability issues that can compromise their long-term effectiveness, necessitating the exploration of alternative, cost-effective, and more sustainable catalysts for advancing electrochemical technologies [144].

Due to the widespread availability of carbon and its favorable conductivity, along with the ability to finely tune its structural and physicochemical properties [145], carbon-based materials have emerged as highly promising catalysts for ORR, OER, and HER [146,147]. Notably, carbon-based catalysts offer advantages over their non-precious transition metal counterparts, including relatively lower cost, reduced heavy metal pollution, and environmental friendliness [148]. Beyond commonly known forms like graphite, diamond, and amorphous carbon, various carbon allotropes exist, encompassing 0D fullerene, 1D CNT, 2D graphene, and 3D graphitic carbons, among others [144]. For instance, graphene exhibits a substantial surface area and excellent electrical conductivity, positioning it as a promising material for electrocatalysis. Its attributes facilitate the effective dispersion of active sites and rapid charge transport, making it particularly advantageous in enhancing catalytic performance [149].

Zhou et al. showed a novel strategy for developing cost-effective metal-free catalysts with exceptional activity and stability for ORR in their work, serving as potential alternatives to carbon-supported platinum catalysts (Pt/C). The approach involves the fabrication of self-constructed carbon nanoparticle (CNP)-coated porous biocarbon derived from the plant moss *Weisiopsis anomala*, a readily available and renewable precursor. The CNPs are self-synthesized and incorporated into the moss-derived carbon matrix through hydrothermal treatment and subsequent carbonization at 900 °C. The resulting CNP-coated biocarbon exhibits a larger surface area compared with CNP-free counterparts. Electrochemical assessments reveal outstanding ORR activity for the CNP-coated biocarbon, with an onset potential of 0.935 V vs. the reversible hydrogen electrode (RHE), closely rivaling commercial Pt/C catalysts (0.962 V vs. RHE) and surpassing CNP-free biocarbon. Additionally, the CNP-coated biocarbons demonstrate high limited current density, long-term stability, and resistance to methanol crossover, outperforming Pt/C [150].

In the work of Huang et al., the significance of biomass-derived carbons through HTC for diverse applications, emphasizing their cost-effectiveness, eco-friendly nature, and suitability for catalysis, is shown. The study introduces a novel approach to HTC, incorporating the biomolecule guanine and various carbohydrates (i.e., glucose, fructose, and cellulose) as carbon precursors, resulting in the formation of 2D crystalline carbonaceous materials. High-temperature carbonization yielded hierarchical porous nitrogen-doped carbons with notable surface areas and nitrogen contents. The resulting 2D carbonaceous materials exhibit exceptional performance in ORR and HER, surpassing Pt-based electrocatalysts [151].

Similarly, the work of Li et al. explores a straightforward method to control the morphology and structure of biomass-derived carbonaceous materials, utilizing the unique cordyceps-like 3D structure of rice husk formed through high-temperature carbonization, with SiC as the main component. The well-organized cordyceps-like SiC structure demonstrates remarkable structural/chemical stability and high electron migration rates, serving as a stable substrate for metal deposition and finding application in electrocatalysis. These natural Si-C composite materials overcome limitations imposed by the intricate internal structure of silicon-rich biomass, offering a new avenue to maximize the utilization of rice husk-based carbon and expanding its application field, including hydrogen production through water electrolysis [152].

**Figure 8:**
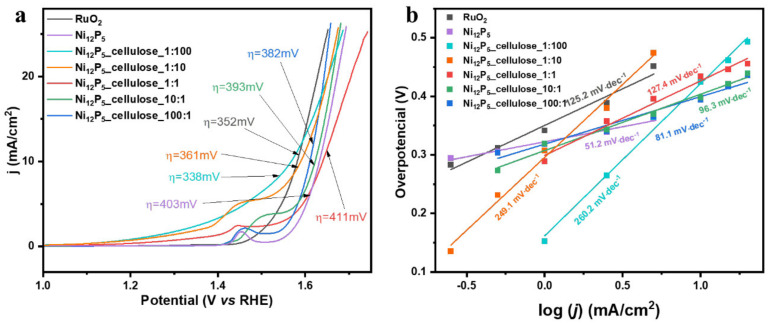
(**a**) LSV (measuring the current response as a function of the applied potential) and (**b**) Tafel plots of RuO_2_, Ni_12_P_5_, and Ni_12_P_5__cellulose composites. Reproduced from [153].

Mijowska et al. investigate hydrogen technology in their study, specifically focusing on nickel phosphide-based electrocatalysts known for their effectiveness in HER and OER. They introduce a simple strategy involving the production of highly porous carbon flakes derived from cellulose fibers, exhibiting exceptional specific surface area (3164 m^2^/g) after activation at 850 °C. The resulting composite of Ni_12_P_5_ and carbon flakes (100:1 ratio) demonstrates superior kinetics for the OER, outstanding durability, and stability under high current density, making it a promising candidate for practical applications [153]. As shown in Figure 8a, the Ni_12_P_5__cellulose_1:100 sample demonstrated the smallest overpotential at 10 mA cm^−2^ current density (η = 338 mV) in comparison with other ratios. The uniform deposition of Ni_12_P_5_ nanoparticles on the carbon sample contributed to the enhanced electrocatalytic activity, highlighting the potential for further modifications for increased effectiveness. Figure 8b shows the Tafel slope, an important parameter indicating electrocatalytic activity. It provides insights into the efficiency of a material in electrochemical reactions. A lower Tafel slope generally signifies a more efficient electrocatalyst. In this study, Ni_12_P_5__cellulose_1:100 displayed the lowest Tafel slope, indicating superior electrocatalytic efficiency compared with other composites. Interestingly, the Tafel slope decreased with increasing Ni_12_P_5_ content, suggesting a change in the rate-determining step and improved reaction kinetics [153].

Despite subjecting biomass-derived carbon to high-temperature carbonization, the resulting material often retains a lower degree of crystallization or graphitization, contributing to a substantial presence of structural disorders. This inherent disorderliness becomes a limiting factor in facilitating fast charge transfer within the carbon framework. Moreover, carbonaceous materials with pronounced disorder tend to exhibit reduced resistance to corrosion in harsh environments, which is a drawback for achieving prolonged catalytic stability [39]. To address these challenges and enhance catalytic performance, transition metal salt catalysts are frequently incorporated during the synthesis process. These catalysts not only contribute to the improvement in crystalline structure but also play a crucial role in introducing and activating more active species within the carbon matrix [144]. This approach aims to overcome the inherent limitations of disorder in biomass-derived carbon, promoting enhanced charge transfer and catalytic stability for various applications.

Biomass-derived carbonaceous materials encounter various challenges. Firstly, biomass often lacks certain heteroatoms, necessitating an additional source, potentially leading to poor material homogeneity [154,155]. Structural control of biomass-derived carbon is challenging, lacking guiding principles for nanostructured design [156]. Variability among materials from the same biomass precursor poses a reproducibility challenge. Impurities in biomass precursors are hard to remove entirely, and their roles in electrocatalysis are unclear. Finally, optimization of the graphitization degree, structure, morphology, and porosity of biomass-derived carbonaceous materials is essential [157].

In conclusion, fuel cells stand as efficient and environmentally friendly devices in converting chemical energy to electricity through electrochemical processes, with HER at the anode and ORR at the cathode playing pivotal roles. The current reliance on precious metal-based catalysts presents challenges, including cost and stability issues, urging the exploration of alternative, sustainable materials. Carbonaceous materials, owing to their abundance, tunability, and favorable conductivity, emerge as promising candidates for ORR, OER, and HER electrocatalysts. The presented studies of carbonaceous materials derived from biomass highlight the potential of these materials in advancing electrochemical technologies. However, challenges persist, such as structural control, material homogeneity, and the need for optimization in biomass-derived carbonaceous materials. Future research should focus on overcoming these challenges and developing more efficient and sustainable electrocatalysts for widespread application in energy conversion technologies.

### 3.6. Microbial Fuel Cell Electrodes

**Figure 9:**
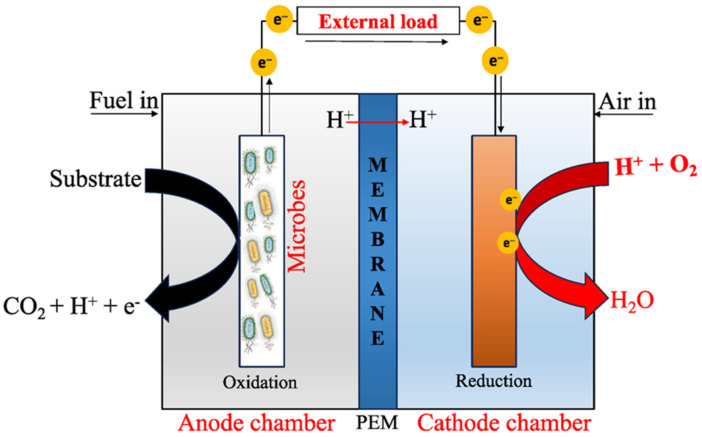
Schematic representation of a typical MFC process. Reproduced from [141].

Fuel cells are devices aimed at converting chemical energy into electrical energy without combustion. Microbial Fuel Cells (MFCs), a type of electrochemical fuel cell, use microorganisms to oxidize organic matter in wastewater. Typically, MFCs include an anode and a cathode separated by a proton exchange membrane like Nafion or poly(tetrafluoroethylene). The bacterial biofilm in the anode acts as a catalyst, transforming organic molecule energy into electrons, while oxygen is reduced at the cathode to form water [158]. Figure 9 shows a schematic representation of a typical MFC.

Designing suitable bio-anodes is crucial to broadening the applications of MFCs. Moreover, the substantial expense associated with components utilized in MFC reactors is a significant constraint, impeding the timely commercial deployment of this technology [62]. Significant advancements in developing highly efficient electrode materials for MFCs have been recently developed. Among the sustainable materials used for this purpose, carbon-based materials are highlighted [159,160].

Carbon-based electrodes are frequently utilized in MFCs owing to their biocompatibility, extended durability, excellent conductivity, and cost-effectiveness. The versatility of carbon materials lies in their ability to manifest diverse morphologies and structures, facilitating the design of attractive and efficient electrodes [161]. In particular, carbon-based anodes play a crucial role in MFCs by promoting the efficient attachment of bacteria. Additionally, these anodes establish a conductive pathway for electron transfer, contributing to the overall efficacy of the MFC system [160,161].

Liu et al. conducted an experiment utilizing eight graphite anodes paired with a single cathode in a single-chambered MFC. They observed a notable 80% decrease in chemical oxygen demand from the initial value using these anodes. However, despite this reduction, the maximum power achieved was approximately 26 mW/m, highlighting a limitation in the use of low-porosity carbon rods within MFCs [162].

To address the issue of low surface area, Lovley et al. successfully employed materials with higher surface areas, such as graphite felt electrodes. This modification led to a threefold increase in the maximum current produced, emphasizing the significance of electrode surface area in MFC performance [163]. Another innovative approach, implemented by Logan et al., utilized a graphite fiber brush electrode wound around a titanium wire to augment the surface area and facilitate microbial inoculation. This modification resulted in a remarkable maximum power density of up to 2400 mW/m, approximately four times higher than that achieved with carbon paper electrodes [164,165]. Additionally, the use of carbon cloth, a material similar to graphite felt, demonstrated a noteworthy maximum power density of up to 483 mW/m [166]. These advancements underscore the importance of optimizing electrode materials and surface areas to enhance the overall performance of MFCs.

Carbon materials exhibiting thin, two-dimensional morphologies, such as carbon paper, cloth, or mesh, hold great promise as anode materials in MFCs. In contrast to the graphite-based anodes discussed earlier, these morphologies offer distinct advantages in minimizing the separation distance between the two electrodes. This reduction in distance not only diminishes the overall dimensions of the MFC device but also holds the potential to enhance MFC performance [167].

The utilization of such materials addresses the challenge of electrode spacing, contributing to the optimization of MFC design. The proximity of the electrodes facilitates more efficient electron transfer and microbial interactions, ultimately improving the overall functionality of MFCs [158].

Carbon nanotubes (CNTs), distinctive allotropes of carbon, are also a highly promising alternative for MFC electrodes. This stems from their remarkable attributes, including unique electrical conductivity, chemical stability, biocompatibility, high specific area, and catalytic properties [168]. Notably, CNTs exhibit strong cell adhesion, cell attachment, and growth properties, as reported in previous studies [169,170].

Recent research by Erbay et al. highlights the exceptional charge transfer characteristics of microbes grown on CNTs, attributed to the stacking interaction between the carbon atoms of graphite and the cellular outgrowths of microorganisms [171]. Growing CNTs directly over a stainless steel mesh helps maintain low ohmic resistance. The observed advantage of spaces between CNTs facilitates microbe inoculation, and the minimal presence of amorphous carbon contributes to excellent charge interaction. In a related development, Tsai et al. coated CNTs onto carbon cloth to create a highly conductive MFC anode with an expanded surface area, resulting in a notable 250 percent improvement in maximum power density [172].

As shown before, CNTs have emerged as one of the most promising electrode materials, owing to their expansive specific surface area, high mechanical strength, ductility, and outstanding stability and conductivity. Notably, recent attention has focused on conductive polymer/CNT composites, recognizing the synergistic effects that arise from incorporating CNTs into conductive polymers [173].

Qiao et al. explored the feasibility of an MFC utilizing a CNT/polyaniline composite as the anode material. Their findings suggested that CNTs could augment the electrode surface area and electron transfer capability. Furthermore, polyaniline, acting as a conductive polymer, not only offered protective benefits to microorganisms but also enhanced the electro-catalytic activity of the catalyst [174].

In another study, Sharma et al. developed an MFC with a carbon paper anode coated with multi-walled CNTs. Surprisingly, they observed a six-fold increase in power density compared with that achieved with a pure graphite electrode. This improvement was attributed to carboxyl groups on the surface of multi-walled CNTs, which heightened the chemical reactivity of metal nanoparticles [175].

Zou et al. employed polypyrrole (PPy)/CNTs as the anode material, revealing superior electrochemical properties of the modified carbon paper [176]. Tsai et al. supported the idea that a modified electrode could enhance MFC performance. In comparison with an unmodified electrode, they observed a remarkable 148% increase in power density and a 147% increase in cell voltage [172]. These studies underscore the versatility and efficacy of CNTs and conductive polymer/CNT composites in advancing the capabilities of MFC anodes.

**Figure 10:**
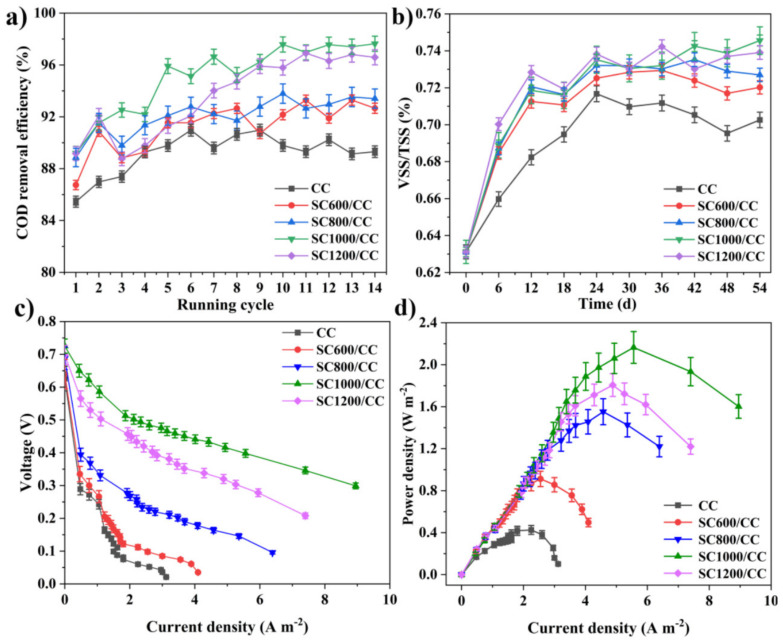
(**a**) The efficiency in COD removal by MFCs; (**b**) the ratio of volatile suspended solids/total suspended solids in the inoculated anaerobic granular sludge within MFC systems; (**c**) polarization curves; and (**d**) the power density of MFCs. Reproduced from [177].

Alternatively, Zhu et al. used sludge-derived carbon-modified material in their work as an anode in an MFC. They prepared different electrodes using several carbonization temperatures. These authors found that MFCs with sludge carbon electrodes, especially those obtained at a carbonization temperature of 1000 °C, exhibited improved performance in chemical oxygen demand removal efficiency, organic matter degradation, and electrochemical oxidation activity compared with carbon cloth electrodes. The sludge carbonization temperature significantly influenced MFC system voltage output, with sludge carbon electrodes consistently outperforming carbon cloth electrodes [177]. This can be seen in Figure 10.

In conclusion, MFCs show promise in converting organic matter into electricity, with bio-anode design being a critical factor. Carbonaceous materials, owing to their biocompatibility, durability, and conductivity, play a pivotal role in enhancing MFC performance. Recent advancements include utilizing materials like graphite felt, carbon cloth, and CNTs to optimize electrode surface area and facilitate efficient electron transfer. Notably, CNTs, either alone or in composite materials with conductive polymers, exhibit exceptional electrochemical properties, offering improved power density and electron transfer capabilities. Future research should focus on further optimizing carbon-based materials, exploring new composites, and understanding microbial interactions to advance the commercial viability of MFC technology. The field holds promise for sustainable energy generation and wastewater treatment, provided ongoing efforts continue to refine and innovate electrode materials in MFCs.

### 3.7. Pollutant Removal

Advanced oxidation processes (AOPs) are delineated as oxidation methodologies wherein hydroxyl radicals (-OH) are generated to effectuate the removal of pollutants from aqueous and gaseous media [178]. These processes offer notable advantages over alternative chemical methods in pollutant treatment, as they exhibit enhanced efficiency and obviate the generation of secondary pollutants [179]. Among the AOPs, ozonation stands out, producing -OH radicals adept at pollutant degradation [180]. Particularly noteworthy are certain metal oxides, such as MnO_2_, Fe_2_O_3_, and MgO, which, when supported on materials featuring developed textural properties, exhibit heightened catalytic activity. In a study conducted by Tian et al., MnO_x_ and FeO_x_ catalysts were meticulously prepared, supported on diverse biochars, for the ozonation treatment of trizine. The favorable porous characteristics of the biochars facilitate pollutant adsorption, and the well-dispersed metal phase enables the exposed surface atoms, typically coated with hydroxyl groups, to manifest as Lewis acids during the ozone decomposition process [181]. The intrinsic characteristics of biochar are pivotal, particularly when it contains catalytically active metal oxides. Chen et al. systematically fabricated biochars derived from refinery sewage sludge characterized by elevated carbon content, Si-O functional groups, and embedded metal oxides capable of catalyzing ozonation through hydroxyl radical generation. Notably, the biochar’s surface oxygenated groups, such as carboxylic acids or lactones, autonomously promote ozone decomposition without necessitating additional support materials [182]. However, the potential leaching of the catalytically active phase raises concerns about secondary contamination, thus instigating a notable interest in the utilization of free-metal catalysts. Cheng et al. addressed this concern by crafting biochar from corn straws and subsequently nitrogen-doping it through urea treatment to modulate its surface properties. The resulting biochar exhibited electron-rich oxygenated groups, conjugated heteroatoms, and defect sites endowed with free electrons, which collectively played a pivotal role in ozone decomposition for the removal of atrazine. The achieved removal rates reached 74%, emphasizing the efficacy of tailored biochar in AOPs for pollutant remediation [183]. This methodology exhibits utility not solely in water pollutant removal scenarios. Cha et al. innovatively synthesized a catalytic system comprising manganese oxides (MnO_x_) supported on biochar for the room-temperature ozone catalytic oxidation of toluene, an unprecedented endeavor in the literature. Their findings revealed a notable efficiency of 90%, albeit without achieving complete oxidation to CO and CO_2_ [184].

**Figure 11:**
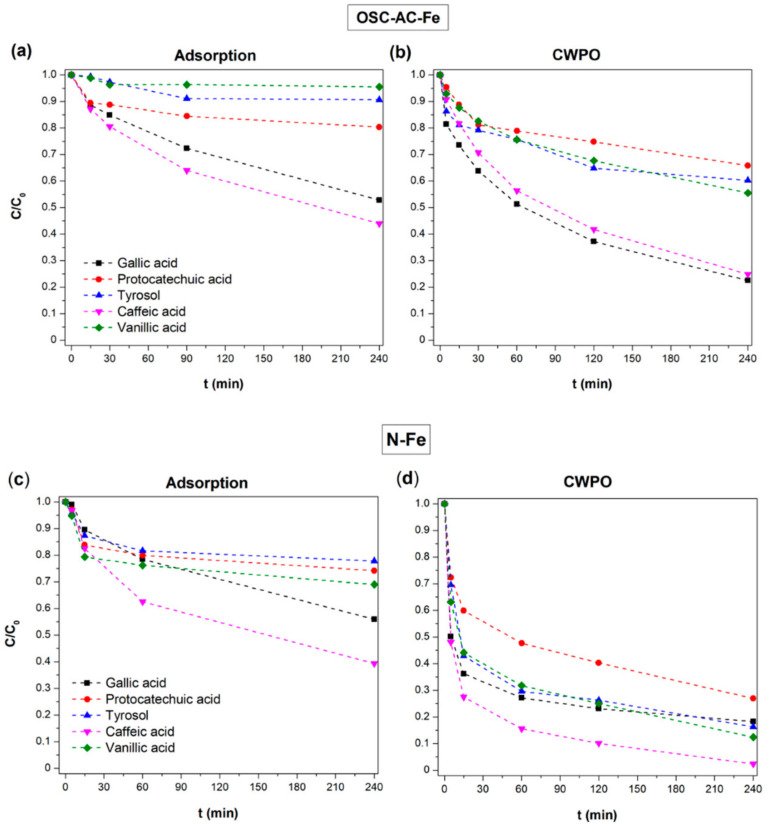
Comparison of phenolic compound removal via adsorption of catalytic wet peroxide oxidation (CWPO) using OSC-AC-FE and N-Fe catalysts: (**a**) Adsorption for OSC-AC-Fe; (**b**) CWPO for OSC-AC-Fe; (**c**) Adsorption for N-Fe; (**d**) CWPO for N-Fe. Reproduced from [186].

An exemplary process known for its efficiency is the Fenton process, predicated on the catalytic capability of ferrous ions (Fe^2+^) to decompose H_2_O_2_ into hydroxyl radicals [185]. Biochars present intriguing potential as supports for Fe catalysts, and Esteves et al. conducted a comprehensive investigation involving the preparation of a series of biochars derived from olive oil industry waste. These biochars were employed for the purification of water contaminated by agro-food facilities. The intricate interactions between phenolic groups and the catalyst surface are contingent upon the porous properties of the biochars and the chemical interplay between the pollutant and the adsorbent surface, specifically surface groups. These factors collectively dictate the catalytic properties of the system. Notably, the prepared biochar-based materials demonstrated exceptional performance, yielding conversions exceeding 90% (Figure 11) [186]. A similar approach was proposed by Jian et al. for the degradation of pyrene, a prevalent polycyclic aromatic compound in water, ultimately achieving total oxidation [187].

An additional crucial process involves the decomposition of peroxymonosulphate (PMS) or peroxydisulphate (PDS) to generate sulfate radicals (SO4•^−^), which typically exhibit high oxidative selectivity and a relatively prolonged lifetime. Although homogeneous transition metals display notable activity, their effectiveness is impeded by challenges associated with separation, and their presence often fails to prevent leaching. As an alternative strategy, various studies have demonstrated the exceptional efficacy of carbonaceous materials in activating PMS and PDS, with biochars, in particular, showcasing commendable adsorption and catalytic degradation capabilities [188]. Functional groups within carbonaceous materials play a pivotal role in influencing adsorptive properties. Consequently, several researchers have undertaken the preparation of biochars derived from biomass for these oxidation processes. For instance, Zhu et al. synthesized biochars from wood for the degradation of clofibric acid using PDS, achieving an impressive 98% conversion within a one-hour timeframe [189]. He et al. fabricated biochars from sugarcane residues characterized by a high surface area and excellent electron transfer ability, resulting in a 100% bisphenol A degradation efficiency within one hour, along with a PMS decomposition efficiency of 58% [190]. In a similar vein, Guo et al. utilized Fe catalysts derived from a mineral with elevated Fe_2_O_3_ content. However, it has been established that the catalytically active species for PDS decomposition is Fe^0^, necessitating stabilization. This is achieved through the co-pyrolysis of biochar sources (solid residues of red mud and coconut bark) with the metal catalyst, culminating in the production of stabilized Fe^0^. Remarkably, this methodology exhibits excellent PDS activation, leading to the complete removal of orange 7 within 30 min [191].

Table 3 provides a summary of diverse solid-base catalysts derived from biomass for AOP.

In conclusion, advanced oxidation processes, notably ozonation and the Fenton process, offer efficient avenues for pollutant removal. Biochars, when tailored with catalytically active metal oxides or used as supports for Fe catalysts, demonstrate exceptional efficacy in these processes. The intrinsic properties of biochar, such as developed textural characteristics and surface oxygenated groups, contribute to enhanced catalytic activity. Additionally, biochars play a crucial role in activating PMS or PDS for sulfate radical generation, showcasing commendable adsorption and catalytic degradation capabilities. Future research should delve into optimizing biochar synthesis, exploring new catalyst–biochar combinations and addressing potential challenges like leaching in AOPs. These developments will contribute to advancing the effectiveness and applicability of biochar-based materials in environmental remediation processes.

## 4. Conclusions

Herein we have reviewed the main advances in the field of using renewable carbonaceous materials in catalytic processes. The studies reviewed confirm the technical viability of the applications proposed in most cases. For biofuel production, biochar-supported catalysts have proved to be able to achieve biodiesel production with yields exceeding 70%. Notably, hydrochars and activated carbons derived from diverse biomass sources have demonstrated significant tar removal efficiency. For instance, rice husk char exhibited an increased BET surface area from 2.2 m^2^/g to 141 m^2^/g after pyrolysis at 600 °C, showcasing its effectiveness in adsorbing phenol and light aromatic hydrocarbons. Concerning chemical production and the oxidation of alcohols, the influence of biochar quantity and pre-calcination temperature on catalytic performance has been proven, achieving selectivity toward benzaldehyde exceeding 70%. In aldehyde oxidation, biochars co-doped with selenium and nitrogen exhibited yields exceeding 90%. When it comes to hydrogenation reactions, biochar-supported Pd catalysts showed total conversion of benzaldehyde to toluene within 2 h, while in the reduction of nitroarenes, RuO_2_ and Pt catalysts on biochar achieved conversions exceeding 97%, with the incorporation of Fe_3_O_4_ enhancing recyclability. In the discipline of photocatalysis, particularly in wastewater remediation, the importance relies on catalysts like TiO_2_. Biochars, when incorporated into TiO_2_ composites, address challenges such as bandgap limitations and electron–hole recombination, enhancing catalytic activity. Renewable CNT–stainless steel mesh electrodes demonstrated exceptional charge transfer, achieving a 250% increase in power density by coating CNTs onto carbon cloth. Furthermore, tailored biochars, rich in surface oxygenated groups, serve as effective catalysts for ozone decomposition, achieving significant pollutant removal rates, exemplified by the removal of atrazine at 74%. All in all, this review paper showcases the importance of renewable carbonaceous materials in the path toward sustainable carbon-based compounds for catalytic applications.

## Figures and Tables

**Table 1:** Carbonaceous materials are obtained from biomass through different treatments and their applications.

Biomass/Waste	Carbonaceous Material	Treatment	Application	Reference
Coconut coir dust	Highly curved graphite structures	HTC + Pyrolysis	Various	[45]
*Cinnamomum camphora* leaves	Graphene	Pyrolysis	Various	[46]
*Perilla frutescens* leaves	O/N-co-doped porous carbon nanosheets	Pyrolysis	Electrode materials for supercapacitors	[47]
Watermelon pulp	Carbon-based composite powder (micrometer particles and nanosheets)	HTC	Anode materials of lithium-ion batteries	[48]
Cellulose	Graphitic carbon nanostructures	HTC + Impregnation	Fuel cell catalytic supports/anodes in Li-ion batteries.	[49]
*Brassica juncea* L. plants	Carbon nanotubes	Extraction + thermal treatment	Photocatalysts	[50]
Sawdust	Nanofibers/mesoporous carbon composites	Catalytic pyrolysis	Electrode materials for electrochemical energy storage	[51]
Spruce bark	Graphene nanosheet arrays	HTC + KOH activation	Electrode material for supercapacitors	[52]
Rice husk	Graphene-like materials	Thermal treatment + KOH activation	Graphene materials to improve cement mortar strength	[53]
Sphagnum moss, corn stalks, cotton, and prickly bamboo	Carbon nanotubes	Pyrolysis + mechanochemical activation	Hydrogen storage	[54]
Cotton	Multilayer carbon nanotubes	Pyrolysis + mechanochemical activation	Adsorbent	[55]
Sugarcane bagasse	Graphene-like nanosheets	Carbonization + KOH activation	Supercapacitors	[56]
Rice straw	Carbon nanotubes	Chemical pretreatment + HTC	Catalyst supports	[57]
Softwood sawdust	Graphitic nanotubes	Chemical pretreatment + pyrolysis	Electrode or filtration applications	[58]
Sugarcane bagasse	Nanostructured biochar	Microwave-assisted pyrolysis	Various	[59]
Wheat straw	Graphene sheets	HTC + graphitization	Anode material for lithium-ion batteries	[60]
Coconut shell	Porous graphene-like nanosheets	Chemical activation + pyrolysis	Supercapacitor	[61]
Almond shell	Biochar	Pyrolysis	Electrode in microbial electrolysis cell	[62]
Peanut dregs	Porous carbon material	Pyrolysis + chemical activation	Multiple energy storage applications	[63]
Sugar cane	Graphene-like material	Pyrolysis + chemical activation	Adsorbent	[64]
Lignin	Layered graphene-like structure	Chemical solution combustion	Adsorbent	[65]

**Table 2:** Comparison of different biochar-derived catalysts.

Reaction	Catalyst	Conditions	Catalytic Efficiency	Ref.
Biodiesel Synthesis	NiO-MoO/biochar	75 °C, 60 min	77% yield	[71]
Biodiesel Synthesis	CaO/biochar	100 °C, 180 min	91% yield	[72]
Biodiesel Synthesis	CaO-SiO_2_/biochar	65 °C, 150 min	94% yield	[73]
Biodiesel Synthesis	Biochar	60 °C, 120 min	98% yield	[74]
Biodiesel Synthesis	Sulfonated biochar	80 °C, 40 min	100% yield	[75]
Biodiesel Synthesis	Sulfonated biochar	65 °C, 60 min	82% yield	[67]
Biodiesel Synthesis	Sulfonated biochar	65 °C, 360 min	99% yield	[76]
FTS	Co/biochar	500 °C, 2 MPa	67% conversion	[79]
FTS	N-doped biochar	300 °C, 2 MPa	92% conversion	[80]
FTS	Fe/biochar	300 °C, 2 MPa	46.7% conversion	[81]
DRM	Biochar	900 W (MW)	75% CH_4_ conversion	[84]
DRM	Biochar	900 °C	65% CH_4_ conversion	[85]

**Table 3:** Comparison of different biochar-derived catalyst performances regarding AOP, catalyst, yield, and removal efficiency.

AOP	Catalyst	Pollutant	Removal Efficiency	Ref.
Ozonation	MnOx/biochar	Trizine	34%	[181]
	FeOx/biochar		35%	
Ozonation	Biochar	Refineries residues	80%	[182]
Ozonation	Biochar	Atrazine	74%	[183]
Ozonation	MnO_x_/biochar	Toluene	90%	[184]
Fenton process	Fe/biochar	Olive mill wastewater	92%	[186]
Fenton process	Fe_3_O_4_/biochar	Pyrene	100%	[187]
PDS activation	Biochar	Clofibric acid	98%	[189]
PMS activation	Biochar	Bisphenol A	100%	[190]
PDS activation	Fe/biochar	Orange 7	100%	[191]

## Data Availability

Not applicable.
